# Supplementary material for: Radiology Access in Rural Germany: A Nationwide Survey on Outpatient Imaging and Teleradiology
Source: Diagnostics (Basel). 2025 Apr 10;15(8):962. doi: 10.3390/diagnostics15080962 (PMC12026352; doi:10.3390/diagnostics15080962)
Supplement: Supplementary file 1 [file diagnostics-15-00962-s001.zip › diagnostics-3465582-supplementary.pdf]

Improving Radiology Access in Rural Areas: Outpatient Imaging and Teleradiology

In this study, teleradiology refers to the remote interpretation of imaging studies by board-certified radiologists via digital transmission. The questionnaire focused on teleradiology modalities commonly practiced in Germany, primarily including computed tomography (CT), plain radiographs (X-rays), and magnetic resonance imaging (MRI). Ultrasound and interventional procedures were not considered relevant for teleradiology, as they typically require on-site performance and direct patient contact.

Outpatient imaging refers to medical imaging procedures (like X-rays, CT scans, MRIs, ultrasounds, etc.) that are performed without requiring the patient to be admitted to a hospital. Instead, the patient comes to a clinic, imaging center, or hospital department for a short visit, has the scan done, and then goes home the same day.

What specialty do you work in?

Please choose...

How many years of professional experience do you have?

#

0 / 80

Please select your field of activity.

- ○ Practice doctor
- ○ Resident physician in a clinic
- ○ Senior physician in a clinic
- ○ Chief physician in a clinic

What is your attitude towards the use of teleradiology?

(1 star rather negative 5 stars very positive)

☆☆☆☆☆

To what extent is teleradiology currently used in your region?

- ○ Widely used

- Moderately used
- Sparsely used
- Not introduced yet

What concerns do you have about teleradiology?

- Missing diagnostic discussions of Radiologists with Patients
- Lack of communication of Teleradiologists with requesting Physicians

  In teleradiology, direct communication between teleradiologists and referring physicians is often missing, as contact typically occurs only through reports or digital means. As a result, important clinical information, such as specific symptoms or the exact context of the examination, may be lost.
- Overdiagnosis due to the expansion of imaging

  The concern is that radiological findings might be overinterpreted when presented without direct clinical context.
  For example: An MRI of the lumbar spine is requested due to nonspecific back pain without neurological deficits. The teleradiologist reports age-related changes, such as small disc protrusions, which are not relevant to the symptoms but could be overinterpreted. This may lead to unnecessary treatments or referrals.
- Data Protection

  In teleradiology, medical imaging data and patient information must be transmitted digitally, posing risks of data breaches, such as inadequately secured networks or cyberattacks. Additionally, there is concern that unauthorized individuals could gain access to sensitive health data if the information is not encrypted or sufficiently anonymized.
- Technical Failures

  Teleradiology relies heavily on stable technical infrastructures. Network outages, server issues, or delays in data transmission can result in imaging data not being transmitted or evaluated in a timely manner. This can delay diagnosis and treatment, particularly in time-sensitive cases.

What role do you think teleradiology could play in improving access to radiology examinations?

- Enhanced radiologist availability for consultations
- Real-time image transmission for remote reporting

- Possibility to get second opinions when radiology report is unclear
- Possibility to obtain expert opinions from subspecialized radiologists

What factors might improve access to rural imaging coverage?

- Offering radiology services in local hospitals

  Local hospitals often offer a limited range of radiological services, such as X-rays or ultrasounds. Expanding radiological services, for example through CT or MRI, could significantly improve healthcare in rural areas.
- Increased number of radiology practices
- Improved teleradiology infrastructure

  It enables radiologists to review imaging remotely and offer specialist opinions

To what extent could outpatient care improve access to radiological examinations in rural areas?

(1 = Negative, 5 = Positive)

☆ ☆ ☆ ☆ ☆

What role could outpatient radiology play on a Patients' waiting time for radiological examinations?

Shorter wait time — Longer wait time

Do you have more trust in radiology reports from a hospital or from a private practice?

Reports from Practices — Reports from Hospitals

Provide reasons why routine radiology services should be performed in a practice rather than in a hospital. (1 most important, 6 least important)

1 = most important
6 = least important

Shorter travel distances (Radiology practices are often located near residential areas, whereas hospitals tend to be centralized)

Patient comfort (practices can often offer a more relaxed environment)

Cost efficiency (lower operational costs compared to hospitals)

Shorter waiting times on site (no emergency care)

Continuity of care (with regular follow-up examinations)

Specialization (radiology practices often specialize in certain imaging techniques or diagnostic procedures)

Faster appointment scheduling (shorter waiting times for routine exams)

1.
